# Methamphetamine Use among Newly Diagnosed HIV-Positive Young Men in North Carolina, United States, from 2000 to 2005

**DOI:** 10.1371/journal.pone.0011314

**Published:** 2010-06-25

**Authors:** Christopher B. Hurt, Elizabeth Torrone, Kelly Green, Evelyn Foust, Peter Leone, Lisa Hightow-Weidman

**Affiliations:** 1 Department of Medicine, Division of Infectious Diseases, School of Medicine, University of North Carolina at Chapel Hill, Chapel Hill, North Carolina, United States of America; 2 Department of Epidemiology, School of Public Health, University of North Carolina at Chapel Hill, Chapel Hill, North Carolina, United States of America; 3 Social and Behavioral Science Research Core, Center for AIDS Research, University of North Carolina at Chapel Hill, Chapel Hill, North Carolina, United States of America; 4 North Carolina Department of Health and Human Services, HIV/STD Prevention and Care Branch, Raleigh, North Carolina, United States of America; Duke University Medical Center, United States of America

## Abstract

**Background:**

Methamphetamine (MA) is a new arrival to the Southeastern United States (US). Incidence of HIV is also increasing regionally, but data are limited regarding any association between this trend and MA use. We examined behavioral data from North Carolina (NC) residents newly diagnosed with HIV, collected by the Department of Health between 2000-2005.

**Principal Findings:**

Among 1,460 newly diagnosed HIV-positive young men, an increasing trend was seen from 2000-2005 in MA use (p = 0.01, total n = 20). In bivariate analyses, users of MA had significantly greater odds of reporting other substance use, including alcohol, powder or crack cocaine, marijuana, and methylenedioxymethamphetamine (MDMA, “ecstasy”). They were also more likely to have reported sexual activity while traveling outside NC; sex with anonymous partners; and previous HIV testing. In a predictive model, MA use had a negative association with nonwhite race, and strong positive associations with powder cocaine, “ecstasy,” or intravenous drug use and being a university student.

**Conclusions:**

Similar to trends seen in more urban parts of the US, MA use among newly diagnosed, HIV-positive young men is increasing in NC. These data are among the first to demonstrate this relationship in a region with a burgeoning epidemic of MA use. Opportunities exist for MA-related HIV risk-reduction interventions whenever young men intersect the healthcare system.

## Introduction

Methamphetamine (MA) use increases risk behaviors for HIV infection among both heterosexual men[1] and those who have sex with other men (MSM) [2], [3]. As with alcohol and traditional street drugs such as heroin or cocaine, impaired judgment and disinhibition of the user may contribute to higher-risk behaviors [4], [5], [6]. MA users typically experience up to 12 hours of heightened alertness, increased energy, and a sense of invulnerability, often accompanied by increased libido [7]. Some MSM who typically are not the receptive partner for anal intercourse may do so because of drug-induced erectile dysfunction[7] – thus placing themselves at significantly increased risk for HIV transmission [8]. Increased pain tolerance, heightened libido, and a sense of endurance lead to “marathon” sex sessions among some users, which can last from hours to days [9].

Use of MA is clearly associated with increased HIV incidence among MSM in major cities of the Pacific coast and Northeastern United States (US) [10], [11], but its impact outside of urban centers and in other regions of the country is much less clear. In particular, data on MA in the Southeastern US are scarce. Similar to the rest of the country, the Drug Enforcement Administration (DEA) acknowledges that clandestine laboratory manufacture of MA in NC diminished following legal restrictions placed on the availability of precursor chemicals, like pseudoephedrine. However, the trafficking of higher-purity MA from Mexico along routes traditionally used for cocaine has resulted in the availability of MA in metropolitan centers of the state – and increasingly in rural areas [12]. Large portions of the region are unsampled by major national drug abuse surveillance networks [13], [14], and despite increasing calls for awareness of MA-associated health risks, the public health response has been limited.

As in most of the Southeast, NC is disproportionately hard-hit by the HIV epidemic; its case rates exceeded the national rate in 2006 [15]. Among HIV cases diagnosed in NC, the proportion among men rose from 66% in 2000 to 73% in 2006; MSM accounted for 25% of cases in 2000 and 36% in 2006 [16], [17]. Little is currently known about MA use as a risk factor for HIV infection among men in NC. The purpose of this study was to determine the extent of substance use among NC's newly diagnosed HIV-positive men from 2000 to 2005, with special attention to MA. Secondarily, we sought to better characterize other risk factors of MA-experienced men in this large cohort.

## Methods

### Participants

Providers and laboratories in NC use a mandatory, confidential, name-based system to report all diagnoses of any stage of syphilis or HIV disease to the state's Department of Health and Human Services (NC-DHHS). Each case is assigned to a Disease Intervention Specialist (DIS), an officer of the NC-DHHS's Partner Counseling and Referral Service who arranges for confidential, voluntary post-diagnosis interviews with each individual and collects standardized demographic and behavioral information. These interviews collect information on demographics (age, race, and geographic location); college or university enrollment status; and travel outside of NC, noting when travel included sexual activity. The DIS documents the subject's history of specific HIV risks in the prior year, including both traditional risk factors (e.g., injection drug use, transactional sex) and non-traditional ones (e.g., use of Internet for sex-partner seeking, recreational drug use). The number and gender of sex partners during a period of HIV infectivity prior to diagnosis are also recorded. Finally, the DIS reviews communicable disease control measures and facilitates partner notification and referrals for treatment or services. With over 90% of all reported HIV cases interviewed (often multiple times), this database contains the most comprehensive statewide behavioral data available for new diagnoses of HIV [18], [19].

### Description of Investigations Undertaken

We used NC-DHHS records to identify all men aged 18 to 30 who were diagnosed with HIV between 2000 and 2005, as part of an ongoing investigation[20] of HIV infection in young men in NC.

Data were abstracted from both the standardized fields and written narratives of the DIS interview records by trained research assistants, using a case abstraction form. Data were entered into an Access database (Microsoft Corporation, Redmond, WA) and de-identified prior to analysis.

Descriptive analyses provided demographic, clinical, and behavioral characteristics of the study sample, including reported drug use. Temporal trends in the data were assessed using two-sided Cochran-Armitage trend tests. After identifying factors potentially associated with HIV and drug use from previous reports in the literature, we conducted bivariate analyses of characteristics associated with MA use and calculated odds ratios (OR) with 95% confidence intervals (CI). Finally, we developed an exploratory multivariate logistic regression model to help define those factors most associated with MA use in the cohort. We used likelihood ratio testing to eliminate non-significant predictor variables from an initial model inclusive of all factors of interest. (Factors of interest were not limited solely to those variables with statistical significance in bivariate comparisons.) Reported p-values are all two-sided, with α = 0.05. All analyses were completed using Stata/IC v 10.1 (Stata Corporation, College Station, TX).

### Ethics

Because of its state-sanctioned role in epidemiological surveillance, our study was exempted from Institutional Review Board (IRB) approval.

## Results

Between 2000 and 2005, a total of 7,011 men with a new diagnosis of HIV were reported in NC, of whom 20.8% were between the ages of 18 to 30 (n = 1,460). Numbers of reported HIV cases among these young men increased each year over the period, rising from 178 in 2000 to 323 in 2005. The median age was 25. Non-whites made up 69.2% of the sample (n = 1,011; 971 black, 40 other); 10.7% were Hispanic. Of these men, 51.3% reported sex only with other men, while 14.9% reported sex with both men and women in year before their infection.

Over the five-year period, a statistically significant trend of increasing MA use was reported, rising from 0.6% of men diagnosed with HIV in 2000 to 3.1% in 2005 (p = 0.01, total n = 20). In the same period, powder cocaine (p = 0.193) and marijuana (p = 0.019) use increased slightly while crack cocaine use fell (p = 0.083). Injection drug use was infrequent and did not change over time (p = 0.689).

In bivariate analyses (Table 1), MA users were significantly less likely to be black (OR 0.22, 95% CI 0.08, 0.60), and tended to have sex with men only (OR 1.81, 95% CI 0.60, 5.48). Men who used MA had significantly greater odds of reporting polysubstance use than non-MA users. The strongest associations were noted for use of powder cocaine (OR 21.0, 95% CI 8.21, 53.7), intravenous drugs (OR 21.5, 95% CI 7.18, 64.3), and methylenedioxymethamphetamine (MDMA, “ecstasy”, OR 20.1, 95% CI 7.25, 56.0). We also noted significantly greater odds of alcohol, crack cocaine, and marijuana consumption among MA users.

**Table 1:** Bivariate analyses of factors associated with methamphetamine use among 1,460 men aged 18–30 diagnosed with HIV in North Carolina, 2000 to 2005.

			Unadjusted odds of reporting methamphetamine use	
	n	No. (%) MA users	OR (95% CI)	*P*
**Age**				
18**–**24	671	9 (1.3)	0.96 (0.40, 2.33)	0.931
25**–**30	789	11 (1.4)	Ref	
**Race**				
Black	971	6 (0.6)	**0.22 (0.08, 0.60)**	**0.003**
Other	40	2 (5.0)	1.90 (0.41, 8.82)	0.411
White	446	12 (2.7)	Ref	
**Gender of sex partner(s)**				
Men and women	218	1 (0.5)	0.41 (0.05, 3.67)	0.424
Men only	750	15 (2.0)	1.81 (0.60, 5.48)	0.297
Women only	358	4 (1.1)	Ref	
**Alcohol use**	790	17 (2.2)	**4.89 (1.43, 16.8)**	**0.012**
**Powder cocaine use**	130	13 (10)	**21.0 (8.21, 53.7)**	**<0.001**
**Crack cocaine use**	87	6 (6.9)	**7.19 (2.69, 19.2)**	**<0.001**
**Marijuana use**	465	11 (2.4)	**2.65 (1.09, 6.45)**	**0.031**
**MDMA** * **use**	36	6 (17)	**20.1 (7.25, 56.0)**	**<0.001**
**Intravenous drug use**	27	5 (19)	**21.5 (7.18, 64.3)**	**<0.001**
**Patron of clubs or bars**	437	10 (2.3)	2.37 (0.98, 5.74)	0.055
**Anonymous sex partners**	438	12 (2.7)	**3.57 (1.45, 8.80)**	**0.006**
**Sex in bars or clubs**	54	2 (3.7)	2.97 (0.67, 13.1)	0.152
**Met sex partners in bars**	290	8 (2.8)	**2.74 (1.11, 6.76)**	**0.029**
**Met sex partners online**	219	6 (2.7)	2.47 (0.94, 6.50)	0.067
**Travel outside NC that included sexual activity**	474	12 (2.5)	**3.18 (1.29, 7.82)**	**0.012**
**Student in college or university**	191	5 (2.6)	2.25 (0.81, 6.26)	0.121
**Coinfected with early syphilis**	90	4 (4.4)	**3.94 (1.29, 12.0)**	**0.016**
**HIV testing history**				
Documented	356	10 (2.8)	**4.10 (1.48, 11.4)**	**0.007**
No previous	857	6 (0.7)	Ref	

*MDMA, methylenedioxymethamphetamine.

Compared with non-users, men who reported MA use had greater odds of reporting sex with anonymous partners and having met sex partners in bars or clubs. The number of reported sex partners did not differ significantly between MA users and non-users, nor were users any more likely to have sex in exchange for drugs or money, or sex with a partner known by them to be HIV-positive. (Data not shown). MA users were more likely to have had sex while traveling outside of NC (OR 3.18, 95% CI 1.29, 7.82). Coinfection with early syphilis (primary or secondary) at the time of HIV diagnosis was more likely among MA users (OR 3.94, 95% CI 1.29, 12.0), and users had greater odds of reporting prior testing for HIV (OR 4.10, 95% CI 1.48, 11.4) than those who did not use MA.

In an exploratory multivariable predictive model (Table 2), MA use was most strongly associated with use of powder cocaine, MDMA, or intravenous drugs, and being a student in a college or university. Nonwhite race was negatively associated with MA use.

**Table 2:** Multivariate analyses of factors associated with methamphetamine use among 1,460 men aged 18–30 diagnosed with HIV in North Carolina, 2000 to 2005.

		Adjusted odds of reporting methamphetamine use	
	n	OR (95% CI)	*P*
**Nonwhite race**	1011	0.37 (0.14, 1.01)	0.053
**Powder cocaine use**	130	**12.6 (4.33, 36.6)**	**<0.001**
**MDMA** * **use**	36	**6.00 (1.90, 19.0)**	**0.002**
**Intravenous drug use**	27	**8.01 (2.23, 28.75)**	**0.001**
**Student in college or university**	191	**5.31 (1.63, 17.4)**	**0.006**

*MDMA, methylenedioxymethamphetamine.

## Discussion

Our study is the first statewide description of MA use among newly diagnosed HIV-positive young men in the Southeastern US, and demonstrates an increasing trend in MA use among young men diagnosed with HIV in NC from 2000 to 2005. Although these cross-sectional surveillance data cannot demonstrate causation, certain findings have implications for public health policy in NC and other states in the region.

MA users in this sample are similar to those described in samples from major cities in previous years. They were often self-identified as MSM[21], [22] and tended to engage in more HIV-associated risk behaviors than their non-using counterparts [23], [24], [25], [26]. They frequented clubs or bars[21] and had anonymous sex partners[27] – often met in a bar, club, or online. Internet sex-seeking was prominent among both users and non-users.

In particular, two findings were noteworthy: coinfection with early syphilis and the HIV testing behavior of MA users. NC is currently experiencing a major syphilis outbreak, and MSM are disproportionately impacted [28]. Studies from urban settings demonstrate MA use as a risk factor for incident syphilis infection[29], [30], and previous work in NC suggests increased sexual risk-taking behavior among stimulant users [31].

Frequent HIV testers may regard this as a method of primary prevention; while acknowledging their high-risk sexual activities, they may use a recent negative HIV test as evidence of their “safety” as a partner for unprotected intercourse [32]. Currently, counselors at HIV testing centers (including sexually transmitted infection (STI) clinics) in NC record very limited information about sexual and injection drug HIV risk behaviors on surveillance forms. Our findings underscore the need to expand the inquiry and to more broadly discuss and record non-injection drug use, the circumstances and environment in which drugs are used, and whether or not sexual activity occurs while intoxicated. More comprehensive drug-use assessments at STI clinics could greatly improve our understanding of the expansion of new drugs like MA in the absence of a robust regional surveillance system for drug use.

While relatively few men in this cohort acknowledged MA use in the period prior to their HIV diagnosis, the proportion who did increased significantly over the five-year study period – the same period over which MA infiltration into NC began in earnest. US DEA annual “busts” of clandestine MA laboratories in NC grew from 14 in 2000 to 322 in 2005, but these numbers do not account for higher-purity “crystal” MA imported via established cocaine trafficking routes from Mexico [12]. Thus, these users with newly diagnosed HIV infection may be sentinels for what we will see in the future, as MA use expands in our state.

The inverse association of black race with MA use is noteworthy in our study, and echoes qualitative data collected elsewhere in the Southeastern US. Community and cultural norms among black drug users favor the use of crack or powder cocaine and fuel a strong suspicion of MA as being an impure and unpredictable drug [33]. This trend should not provide a false sense of security, however; although our total number of MA users was small, a third of them were black.

Travel that included sexual activity was associated with MA use in our analyses. Since almost 75% of our sample self-identified as either MSM or having sex with both men and women, many of these young men may intentionally explore their sexual identities away from the close-knit, small communities in which they live. Studies of MSM in San Francisco demonstrated increased HIV-associated risk behavior when traveling away from home for weekend-long dance (“circuit”) parties [34]. In the case of MA, drug availability may also be an issue; the small numbers of users identified in our sample suggests that the drug had yet to widely disseminate among young men in NC by 2005.

Our results have several limitations. Only a small percentage of the entire cohort were MA users. Data are self-reported by individuals being interviewed by an officer of the NC-DHHS, and thus social desirability bias has likely resulted in underestimation of the prevalence of both drug use and sexual risk behavior. Data collection bias may have improved the capture of information on MA use toward the end of the period, as awareness of expanded availability and use of the drug diffused through to more DIS officers. Additionally, ascertainment bias may have been introduced through the use of chart abstraction, although those reviewing the charts were trained for the task. Finally, our data pertain only to men aged 18 to 30, so we cannot characterize MA use or associated factors in men over age 30 in NC.

In summary, the use of MA among young men newly diagnosed with HIV underscores the fact that MA use exists in the Southeast US, and may be expanding among groups at high risk for HIV acquisition and transmission. HIV risk-reduction messages for MA users should be directed at young, college-aged men, including MSM – with special attention to any point of intersection with the healthcare system that includes STI or HIV testing.
